# Biocontrol of *Aspergillus* Species on Peanut Kernels by Antifungal Diketopiperazine Producing *Bacillus cereus* Associated with Entomopathogenic Nematode

**DOI:** 10.1371/journal.pone.0106041

**Published:** 2014-08-26

**Authors:** Sasidharan Nishanth Kumar, Sreerag Ravikumar Sreekala, Dileep Chandrasekaran, Bala Nambisan, Ruby John Anto

**Affiliations:** 1 Division of Crop Protection/Division of Crop Utilization, Central Tuber Crops Research Institute, Sreekariyam, Thiruvananthapuram, India; 2 Department of Botany, SD College, Kalarcode, Thookkukulam, Alappuzha, India; 3 Integrated Cancer Research Program, Division of Cancer Research, Rajiv Gandhi Centre for Biotechnology, Thiruvanathapuram, India; Leibniz Institute for Natural Products Research and Infection Biology- Hans Knoell Institute, Germany

## Abstract

The rhabditid entomopathogenic nematode associated *Bacillus cereus* and the antifungal compounds produced by this bacterium were evaluated for their activity in reducing postharvest decay of peanut kernels caused by *Aspergillus* species in *in vitro* and *in vivo* tests. The results showed that *B. cereus* had a significant effect on biocontrol effectiveness in *in vitro* and *in vivo* conditions. The antifungal compounds produced by the *B. cereus* were purified using silica gel column chromatography and their structure was elucidated using extensive spectral analyses. The compounds were identified as diketopiperazines (DKPs) [cyclo-(L-Pro-Gly), cyclo(L-Tyr-L-Tyr), cyclo-(L-Phe-Gly) and cyclo(4-hydroxy-L-Pro-L-Trp)]. The antifungal activities of diketopiperazines were studied against five *Aspergillus* species and best MIC of 2 µg/ml was recorded against *A. flavus* by cyclo(4-hydroxy-L-Pro-L-Trp). To investigate the potential application of cyclo(4-hydroxy-L-Pro-L-Trp) to eliminate fungal spoilage in food and feed, peanut kernels was used as a food model system. White mycelia and dark/pale green spores of *Aspergillus* species were observed in the control peanut kernels after 2 days incubation. However the fungal growth was not observed in peanut kernels treated with cyclo(4-hydroxy-L-Pro-L-Trp). The cyclo(4-hydroxy-L-Pro-L-Trp) was nontoxic to two normal cell lines [fore skin (FS) normal fibroblast and African green monkey kidney (VERO)] up to 200 µg/ml in MTT assay. Thus the cyclo(4-hydroxy-L-Pro-L-Trp) identified in this study may be a promising alternative to chemical preservatives as a potential biopreservative agent which prevent fungal growth in food and feed. To the best of our knowledge, this is the first report demonstrating that the entomopathogenic nematode associated *B. cereus* and cyclo(4-hydroxy-L-Pro-L-Trp) could be used as a biocontrol agents against postharvest fungal disease caused by *Aspergillus* species.

## Introduction

*Aspergillus* is a filamentous fungus that produces mycotoxins in many food and feed crops. Aflatoxins are toxic, carcinogenic, immunosuppressive, mutagenic, and polyketide-derived secondary metabolites produced mainly by certain strains of *Aspergillus flavus* and *A. parasiticus* and in a less extent by several strains of *A. nomius, A. pseudotamarii, A. bombycis,* and *A. ochraceoroseus* [1]. *A*. *flavus* and *A. fumigatus* are the main source of aflatoxins, the most important mycotoxins in the world’s food supplies. Aflatoxin B1 (AFB1) and B2 (AFB2) are the most important among 18 different types of aflatoxin in crops and agricultural commodities [2]–[3]. Aflatoxins are produced on peanuts, corn, sorghum, cottonseed, pistachio nuts, copra, cereals, fruits, oilseeds, dried fruits, cocoa, and spices in the field and during storage. Aflatoxins occur mainly in hot and humid regions where high temperature and humidity are optimal for moulds growth and toxins production [4]–[5]. Their presence is enhanced by factors such as stress or damage to the crop due to drought before harvest, insect activity, soil type and inadequate storage conditions [6]. Among naturally occurring aflatoxins, AFB1 is considered as the most potent hepatotoxic and hepatocarcinogenic chemical. AFs have been detected in numerous agricultural commodities [7]. Consumption of aflatoxins contaminated foods and feeds are a serious problem from the viewpoint of not only public health, but also economic losses.

Peanuts (*Arachis hypogaea*) is one of the most important food and oilseed crops cultivated and utilized in most parts of the world. They are widely accepted as an excellent source of nutrition to both human and animals due to their high protein content. Several investigations have listed a large number of fungi which could be isolated from peanuts during storage [8]. *A. flavus* is the dominant storage fungus colonizing peanuts, capable of causing seed rots, moulding of seeds, pre- and post-emergence damping off, and reducing seed viability and seedling growth in peanuts [9]. World-wide occurrence of *A. flavus* and aflatoxins in a great variety of food crops especially in nuts has triggered much research with regard to its causes, progress and prevention [3].

The need for protection of food and feedstuffs against *Aspergillus* species is universally recognized and several approaches such as treatment of peanuts with fungicides, fumigants, and plant products have been suggested. Recently there has been an extensive search for alternatives to chemical fungicides that would provide satisfactory *Aspergillus* control with low impact on the environment and human health [10]. Although the use of synthetic fungicides is a most effective decay control treatment, there is an urgent need to find effective and safe non-fungicide means of controlling postharvest pathogens mainly because of the toxicity of the synthetic fungicide residues to humans and the environment [11]. In recent years, some antagonist microorganisms have been applied in biocontrol of postharvest diseases of agricultural products. *Saccharomyces cerevisiae* and lactic acid bacteria (LAB) represent unique groups, which are widely used in food preservation [12].

In the course of studies on entomopathogenic nematode (EPN), a new EPN belonging to the genus *Rhabditis* and subgenus *Oscheius* was isolated from sweet potato weevil grubs collected from Central Tuber Crops Research Institute (CTCRI) farm, Thiruvananthapuram, Kerala, India [13]. A specific *B. cereus* was associated with EPN, which was pathogenic to various agriculturally important insects [13]. Based on molecular characteristics, *Rhabditis (oscheius)* sp. resembles *Rhabditis* isolate at D2 and D3 (nucleotide sequence region) expansion segments of 28S rDNA [13]. The cell free culture filtrate of *B. cereus* was found to inhibit several pathogenic bacteria, fungi and a plant parasitic nematode (*Meloidogyne incognita*) [14], suggesting that it could be a rich source of biologically active compounds. Recently we reported the antifungal activity of the crude extract obtained from a modified liquid medium against *Penicillium expansum* and *Candida albicans* [15]. This modified media is superior to TSB for the production of secondary metabolites by *B. cereus.*

The potential of *Bacillus* species to produce antibiotics has been recognized for past 50 years. *Bacillus* species produce structurally diverse classes of secondary metabolites, such as lipopeptides, polypeptides, cyclic dipeptides, macrolactones, fatty acids, polyketides, lipoamides, and isocoumarins [16]–[17]. These structurally versatile compounds exhibit a wide range of biological activities, including antimicrobial and anticancer effects [16]–[17]. As *Bacillus* strains rapidly grow in liquid media even under stressful conditions and readily forms resistant spores, it might be useful as an effective biocontrol agent against various phytopathogens [18]. Structures, synthesis, and specific functions of diverse antibiotics produced by *B. subtilis* have elaborately been reviewed [19].

The present manuscript deals with the biocontrol property of *B. cereus* in controlling *Aspergillus* species, in peanut food model. Moreover the manuscript also deals with the purification and structural elucidation of the antifungal compounds produced by *B. cereus* in modified liquid medium and also aimed to determine the ability of the isolated compounds in preventing the growth of *Aspergillus* in peanut food model system.

## Materials and Methods

### Chemicals and media

All the chemicals used for extraction and purification were of analytical grade. High performance liquid chromatography (HPLC) grade methanol and thin layer chromatography (TLC) sheets were from Merck Limited, Mumbai, India. Various microbiological media used in the study were from Hi-Media Laboratories Limited, Mumbai, India. Chemical used for preparing the fermentation medium was purchased from SRL Laboratories Ltd., Mumbai, India. Chemicals used for antimicrobial and cytotoxicity assays were purchased from Sigma-Aldrich, USA. All other reagents were of analytical grade and the other chemicals used in this study were of highest purity. The software used for drawing chemical structure was Chemsketch Ultra, Toranto, Canada.

### Test fungal strains

Five *Aspergillus* species used in the present study were *Aspergillus flavus* MTCC 277, *Aspergillus niger* MTCC 282, *Aspergillus tubingensis* MTCC 2425, *Aspergillus fumigatus* MTCC 3376 and *Aspergillus parasiticus* MTCC 2796. Other test fungi used were *Cryptococcus gastricus* MTCC 1715, *Candida albicans* MTCC 3017, *Candida tropicalis* MTCC184, *Trichophyton rubrum* MTCC 296, *Fusarium oxysporum* MTCC 284, *Rhizoctonia solani* MTCC 4634, and *Penicillium expansum* MTCC 2006. All the fungal strains were purchased from Microbial Type Culture collection Centre, IMTECH (Institute of Microbial Technology), Chandigarh, India and were grown on potato dextrose agar (PDA) at 30°C for 3 days and stored at 4°C for further studies.

### Peanut kernels

Peanut kernels with a commercial level of maturity were used immediately after harvest, or stored at 4°C for no longer than 48 h before using. Before treatments, the peanut kernels were washed with tap water, then surfaced-disinfected with 0.1% sodium hypochlorite for 1 min, cleaned with sterile water, and air dried prior to wounding.

### *B. cereus*: Antifungal compounds producing bacteria

The antifungal compound producing *B. cereus* was isolated from 3^rd^ stage infective juveniles of the nematode sample collected from sweet potato weevil grubs. The strain was identified as *B. cereus* (Accession No. HQ200404) based on 16S rDNA and BLAST analysis. The strain was currently deposited in IMTECH and the accession number is MTCC 5234.

### *In vitro* antifungal assay of *B. cereus* against *Aspergillus* species

To evaluate the interactions between the *B. cereus* and the *Aspergillus* species in culture, 6-mm diameter plugs were cut from 4-day-old PDA cultures of *A. flavus* and *A. niger* and then placed on another PDA plate (15 ml/plate) seeded with 1.0 ml of different treatments. The treatments used for the study were as follows: (A) autoclaved modified liquid medium alone; (B) bacterial cell free culture filtrate of modified liquid medium; (C) 1×10^8^ CFU/ml *B. cereus* cell suspension; and (D) control (sterile distilled water). Autoclaved cultures were prepared by autoclaving modified liquid culture broth for 20 min at 121°C. Bacterial cell free culture filtrates were prepared by filtering the supernatant of centrifuged culture of the *B. cereus* through a 0.22 µm membrane filter after 48 h fermentation [20]. Bacterial cells from 72 h cultures were adjusted to 1×10^8^ CFU/ml by adding additional culture filtrate. After inoculation the plates were incubated for 7 days at 37°C. Fungal growth was recorded after 8 days. Growth inhibition was calculated as the percentage of inhibition of radial growth relative to the control [21]. Three replicates were used per treatment and the experiments were repeated twice.

### Efficacy of *B. cereus* on control of *Aspergillus* species *in* *vivo*

Peanut kernels were wounded (5 mm diameter and approximately 3 mm deep) using a sterile borer. Then the peanut kernels were treated with 25 µl of one of the following treatments: (A) autoclaved modified liquid medium alone; (B) bacterial cell free culture filtrate of modified liquid medium; (C) 1×10^8^ CFU/ml *B. cereus* cell suspension; and (D) control (sterile distilled water). Two hours later, 10 µl of *A. flavus* and *A. niger* spores at 5×10^4^ spores/ml was inoculated into each peanut kernels. The peanut kernels were placed in BOD incubator and incubated at 28°C. The number of peanut kernels that were infected with *Aspergillus* species was recorded after 7 days inoculation. There were three replicates of 20 peanut kernels per treatment and the experiment was conducted twice.

### Effect of different incubation times of *B. cereus* on control of *Aspergillus* species *in* *vivo*

The peanut kernels were wounded as described above. 20 µl of 1×10^8^ CFU/ml cell suspension of *B. cereus* that had been grown in nutrient broth for 12, 24, 36, 48, 60 and 72 h was respectively inoculated into the wounds of the peanut kernels, and sterile distilled water was used as control. After 2 h, 10 µl of 5×10^4^ spores/ml suspension of *A. flavus* and *A. niger* was inoculated into each wound. The peanut kernels were placed in a BOD incubator as described above.

### Effect of different concentrations of *B. cereus* on control of *Aspergillus* species *in* *vivo*

The peanut kernels were wounded as described above. The *B. cereus* cell suspensions were adjusted to concentrations of 1×10^6^, 1×10^7^, 1×10^8^, 1×10^9^ and 1×10^1^°CFU/ml with sterile distilled water, respectively. 20 µl of 1×10^6^, 1×10^7^, 1×10^8^, 1×10^9^ and 1×10^1^°CFU/ml cell suspension respectively was inoculated into each wound, and sterile distilled water was used as control. After 2 h 10 µl of 5×10^4^ spores/ml suspension of *A. flavus* and *A. niger* was inoculated to each wound. Treated peanut kernels were incubated in a BOD incubator as described above.

### Isolation, purification and characterization of antifungal compounds produced by *B. cereus*

#### Preparation of crude antifungal substance by fermenting *B. cereus*

The bacterial fermentation was carried out using modified liquid medium. The liquid medium was composed of (g/l): fructose, 10.0; beef extract, 10.0; K_2_HPO_4_, 1.0; KH_2_PO_4_, 1.0; MgSO_4_, 1.0; NaCl, 2.0; Na_2_SO_4_, 1.0. The medium pH was adjusted to 7.0 before autoclaving using NaOH or HCl solution. After preparing the liquid medium, *B. cereus* was inoculated into the flasks containing 100 ml sterile liquid media. The flasks were incubated in an orbital shaker (Remi, Mumbai, India) (120 rpm) at 30°C in dark for 24 h. When the optical density of the culture at 600 nm was approx 1.7, the bacterial cultures were transferred aseptically into 400 ml sterile medium and incubated in the orbital shaker at 30°C in dark for 96 h. The culture media were then centrifuged (10,000 *g,* 20 min, 4°C) followed by filtration through a 0.22 µm filter, to obtain cell free culture filtrate. Twenty litres of cell free culture filtrate were neutralized with concentrated hydrochloric acid and extracted with an equal volume of ethyl acetate thrice. The ethyl acetate layers were combined, dried over anhydrous sodium sulphate, and concentrated at 30°C using a rotary flash evaporator (Buchi, New Castle, USA). The initial antifungal activity of the crude extract was tested against *A. flavus* by disc diffusion method.

### Purification of antifungal compounds

The browny yellow residue (6.38 g) obtained after drying was loaded on a silica gel column (30×600 mm) previously equilibrated with hexane and eluted successively with 200 ml of 100% hexane, 200 ml of linear gradient hexane: dichloromethane (95∶5 to 5∶95 v/v), 200 ml of 100% dichloromethane, 200 ml of linear gradient dichloromethane:ethyl acetate (95∶5 to 5∶95 v/v), 200 ml of 100% ethyl acetate and finally with 200 ml of 100% methanol. About 88 fractions measuring 100 ml each were collected.

Purity of the compounds was tested by TLC (20×20 cm, precoated silica gel 60 GF254 plates) and HPLC. The HPLC analysis was performed using a Shimadzu LC-10AT liquid chromatography (LC; Shimadzu, Singapore) equipped with a quaternary pump, a degasser, an autosampler, a thermostated column compartment, and a variable wavelength detector. Separations were carried out with isocratic elution on a C18 reversed-phase column (5 µm, 4.6×250 mm, Shimadzu, Singapore) connected with a C18 security guard column (3 mm ID×4 mm, Phenomenex, Torrance, USA). The mobile phase consisted of 100% HPLC grade MeOH. The injection volume was 15 µl and detection wavelength was 240 nm. The flow rate was 1 ml/min, and the column temperature was set to 25°C and run time was 10 min for each sample.

### Structure determination of antifungal compounds

Optical rotation of the compounds was measured on a Rudolph Research Autopol III polarimeter (Hackettstown, NJ, USA). UV spectra were measured on a Shimadzu UV-VIS spectrophotometer UV-2450 (Shimadzu, Japan). Nuclear magnetic resonance (NMR) spectroscopy (Bruker DRX 500 NMR instrument, Bruker, Rheinstetten, Germany) equipped with a 2.5-mm microprobe. NMR Spectrometer using DMSO-d6 was deployed to measure ^1^H and ^13^C. All spectra were recorded at 23°C. Chemical shifts are reported relative to the solvent peaks. (DMSO-d6∶^1^H δ 2.50 and ^13^C δ 39.51). High-resolution mass spectrophotomer (HRMS) was performed on a Thermo Scientific Exactive Mass Spectrometer (Thermo Fisher Scientific, Bremen, Germany) with an electrospray ionization mode. The melting point of the pure compounds was measured with a differential scanning calorimeter in a Mettler Toledo DSC 822e instrument (Mettler-Toledo, Schcoerfenbach, Switzerland). Temperature ranges from 30°C to 300°C were employed.

### Absolute configuration determination of compounds by the HPLC analysis of Marfey’s derivatives

A solution of four compounds (1.5 mg) in 6 M HCl (1 ml) was heated to 125°C for 24 h. The dried aqueous solution was resuspended in H_2_O (100 µl), and 1% Marfey’s reagent in acetone (200 µl) and 1 M NaHCO_3_ (40 µl) were added. The mixture was heated at 40°C for 1 h in a shaking water bath, after which it was removed and cooled. The mixture was quenched with 2 M HCl (20 µl), dried, and dissolved in MeOH for HPLC analysis. Amino acid standards (1 mg each) were derivatized with Marfey’s reagent in same manner similar as described above. The HPLC analysis was performed using two solvent systems: (Shimadzu LC-20AD, C_18_ column; 5 µm, 4.6×250 mm; 1.0 ml/min) at 30°C using the following gradient program: solvent A, water +0.2% TFA; solvent B, MeCN; linear gradient 0 min 25% B, 40 min 60% B, 45 min 100% B; UV detection at 340 nm [22].

### Antifungal assay

#### Minimum inhibitory concentration (MIC)

The MIC was performed by broth microdilution methods as per the guidelines of Clinical and Laboratory Standard Institute (CLSI) (formerly, the National Committee for Clinical Laboratory Standards) [23]–[24], with RPMI 1640 medium containing L-glutamine, without sodium bicarbonate and buffered to pH 7.0. Twofold serial dilutions of the test compounds were prepared in media in amounts of 100 µl per well in 96-well U-bottom microtiter plates (Tarson, Mumbai, India). The test fungal suspensions were further diluted in media, and a 100 µl volume of these diluted inoculums was added to each well of the plate, resulting in a final inoculum of 0.5×10^4^ to 2.5×10^4^ CFU/ml for *Candida* species and 0.4×10^4^ to 5×10^4^ CFU/ml for other fungi. The final concentration of test compounds ranged from 1 to 1000 µg/ml. The medium without the test compounds was used as a growth control and the blank control used contained only the medium. Amphotericin B served as the standard drug control. The microtiter plates were incubated at 35°C for 48 h for *Candida* species and 30°C for 72 h for other fungi. After incubation OD600 nm was measured using a microplate reader, and the MIC was defined as the lowest concentration of the antifungal agents that prevented fungal growth.

### Antifungal assay by disc diffusion technique

Compounds were screened for their antifungal activity against test fungi by disc diffusion method [25]–[26]. The fungal cultures were grown on potato dextrose broth. The mycelia mat of fungi of 7-day old culture was suspended in normal saline solution and test inoculum was adjusted to 1×10^5^ CFU/ml. Inocula (0.1 ml) were applied on the surface of the PDA plate and spread by using a cotton swab. Subsequently, filter paper discs (6 mm in diameter) containing MIC concentration of test compounds were placed on the agar plates and incubated at 35°C for 24–48 h. Afterwards, the diameter of the inhibition zone was measured.

### The antifungal compound from *B. cereus* as biopreservative agent in peanut food model system

Eight peanuts kernels (approximately 8.1 g) were soaked in 50 ml of distilled water for 5 h, and then autoclaved at 121°C for 20 min. The autoclaved peanut kernels were soaked in crude ethyl acetate extract of modified medium and pure compound for 8 h at room temperature and then transferred to Petri dishes. Cooked peanuts soaked in methanol and modified medium alone were used as controls. A spore suspension of *A. flavus* and *A. niger* was prepared (10^7^ spores/100 µl distilled water); and approximately 10 µl of the spore suspension of *A. flavus* and *A. niger* was spread on peanut kernels. The inoculated peanut kernels were incubated for 2 days at 30°C, and the fungal growth was examined every day for 10 days [27].

### Cytotoxicity test

The MTT (3-(4, 5-dimethyl thiazol-2-yl)-2, 5-diphenyl tetrazolium bromide) assay was used to determine the cytotoxicity of diketopiperazine. FS normal fibroblast and VERO cells were used for testing. The cell lines were purchased from National Centre for Cell Science, Pune, India and maintained in Dulbecco’s Modified Eagle Medium (DMEM) supplemented with 10% FBS with antibiotics and antimycotics at 37°C in a CO_2_ incubator. Briefly, cells (3×10^3^/well) were seeded in 0.2 ml of the medium (DMEM with 10% PBS) in 96 well plates, treated with drugs for 72 h. and after incubation, cytotoxicity was measured. For this after removing the drug containing media, 25 µl of MTT solution (5 mg/ml in PBS) and 75 µl of complete medium were added to wells (untreated and treated) and incubated for 2 h. At the end of incubation MTT lysis buffer was added to the wells (0.1 ml/well) and incubated for another 4 h at 37°C. At the end of incubation, the optical densities at 570 nm were measured using a plate reader (Bio-rad ELISA reader 680, California, USA). The relative cell viability in percentage was calculated (A _570_ of treated sample/A _570_ of untreated sample ×100) [28].

### Statistical analysis

All statistical analyses were performed with SPSS (Version 17.0; SPSS, Inc., Chicago, IL, USA). Statistical significance was defined as *p*<0.05. Data for disc diffusion assay was presented as means ± standard deviations.

## Results

### Efficacy of *B. cereus* on the growth of *Aspergillus* species *in* *vitro*

In *in* ****** *vitro* tests, different treatments exhibited different inhibitory effects on *Aspergillus* species. The percentage of inhibition of culture filtrate and cell suspension (1×10^8^ CFU/ml) against *A. flavus* was 26 and 20, respectively and whereas for *A. niger* it was 29 and 22, respectively (Table 1). Sterile distilled water and autoclaved modified medium alone did not inhibit the growth of *Aspergillus* species, because the fungal growth reached the edge of the Petri plates.

**Table 1:** Effect of *B. cereus* and cell free culture filtrate on *Aspergillus* species.

Test fungi	Percentage of inhibition (%)			
	Modified liquid medium alone	Cell free culture filtrate	1×10^8^ CFU/ml *B. cereus* cell suspension	Water control
*A. flavus*	+	26±1.72^a^	20±1^b^	+
*A. niger*	+	29±1^a^	22±1.52^b^	+

Values followed by different letters in same row were significantly different according to Duncan’s multiple range test *p* = 0.05.

+ full growth up to the edge of the Petri plate.

### Efficacy of *B. cereus* on control of decay caused by *Aspergillus* species *in* *vivo*

**Figure 1:**
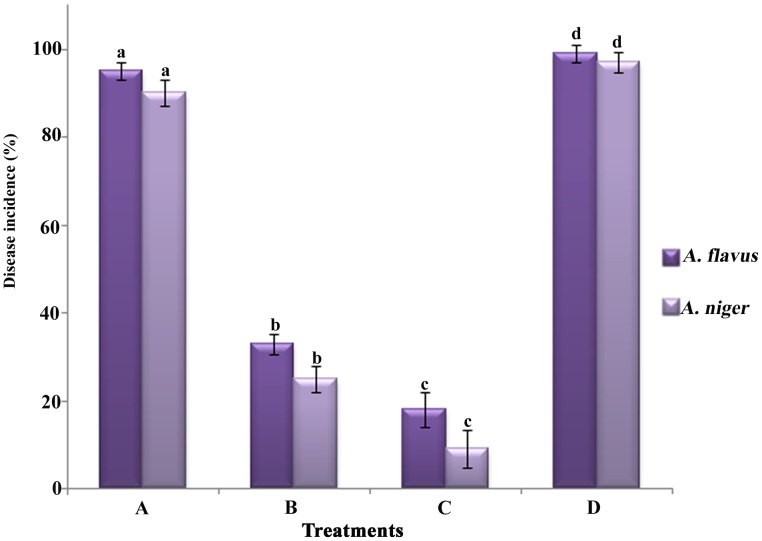
Effect of *B. cereus* on control of *Aspergillus* species. (**A**) autoclaved modified liquid medium; (**B**) cell free culture filtrate of modified liquid medium alone; (**C**) 1×10^8^ CFU/ml *B. cereus* cell suspension; and (**D**) sterile distilled water as a control. Values followed by different letters were significantly different according to Duncan’s multiple range test *p* = 0.05.

As shown in Fig. 1, the cell suspension of *B. cereus* was the most effective treatment on control of the rots in peanut kernels caused by *Aspergillus* species. After 7 days at 28°C, the disease incidence of the water control and autoclaved culture were above 95% for both fungi, whereas the treatment with the bacteria cell suspension at 1×10^8^ CFU/ml was 18% for *A. flavus* and 9% for *A. niger*, which was significantly lower than the treatments with the autoclaved culture, and water control. Meanwhile, the average percentage of the disease incidence in the treatment with the cell free culture filtrate was 33 and 25%, which also recorded remarkable decay control compared with the treatments of water control, autoclaved media alone.

### Effect of different incubation times of *B. cereus* on control of rot caused by *Aspergillus* species *in vivo*

**Figure 2:**
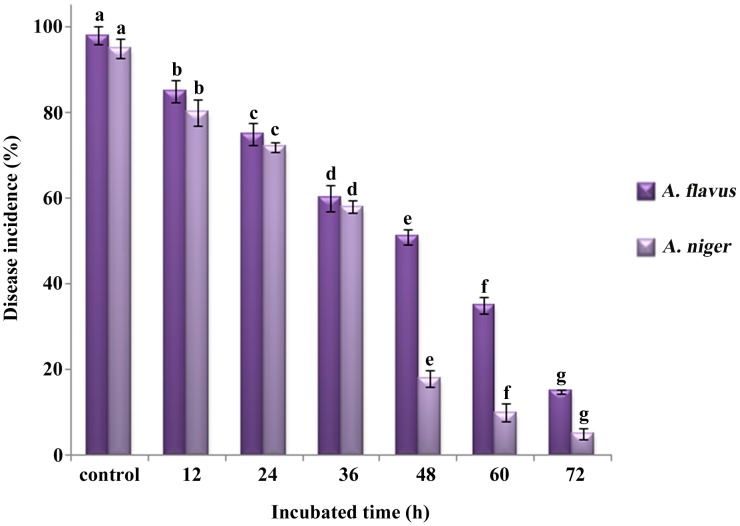
Effect of different incubation times of *B. cereus* on control of *Aspergillus* species. The control is treated with sterile distilled water. Values followed by different letters were significantly different according to Duncan’s multiple range test *p* = 0.05.

Different incubation times of *B. cereus* significantly influenced the effectiveness of the disease control in peanut kernels at 28°C. The results recorded that better biocontrol was obtained when longer incubation time of *B. cereus* was applied (Fig. 2). When the incubation time of *B. cereus* was 72 h, the rate of decay declined to 15% and 5% for *A. flavus* and *A. niger*, respectively. So the best control effect was obtained by the cell suspension of *B. cereus* when the incubation time was 60–72 h.

### Effect of different concentrations of *B. cereus* on control of rot caused by *Aspergillus* species *in vivo*

**Figure 3:**
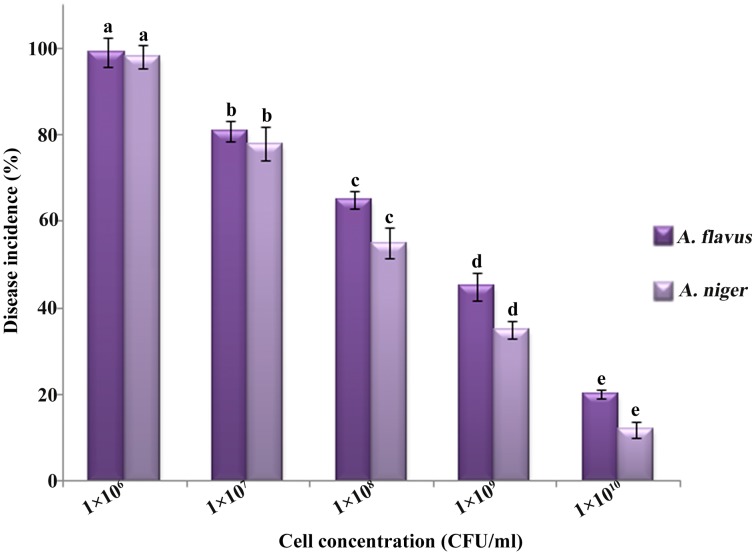
Effect of different concentrations of *B. cereus* on control of *Aspergillus* species. The control is treated with sterile distilled water. Values followed by different letters were significantly different according to Duncan’s multiple range test *p* = 0.05.

The disease incidence on *B. cereus* treated kernels (treated with 20 µl of 1×10^5^, 1×10^6^, 1×10^7^ and 1×10^8^ CFU/ml cell suspension, respectively) was significantly lower than the control. The concentrations of *B. cereus* significantly influenced disease incidence on peanut kernels (Fig. 3). The results showed that the lower disease incidence was recorded when the concentrations of *B. cereus* increases. When the concentration of *B. cereus* was 1×10^8^ CFU/ml and spore suspension of *A. flavus* and *A. niger* was at 5×10^4^ cells/ml, the rate of decay in peanut kernels declined to 20% and 12%, respectively.

### Isolation and purification of antifungal compounds

The crude ethyl acetate extract of the cell free culture filtrate of the *B. cereus* recorded significant antifungal activity against *A. flavus*, the initial test fungus. Silica gel column chromatography of the crude ethyl acetate extract yielded four bioactive fractions, which recorded significant antifungal activity and these fractions were further purified by crystallisation using benzene and hexane to yield four crystal compounds. The column solvent, yield and UV absorption of the pure compounds were shown in the Table 2. Initial bioactivity of the pure compounds was confirmed by testing against *A. flavus.*

**Table 2:** Details of the pure compounds.

S.No	Compounds	Column solvent	Yield (mg)	UV absorption (nm)	Melting point (°C)	HPLC Retention time (min)	Optical rotation (c, 0.02, MeOH)
1	Cyclo-(L-Pro-Gly)	30% DCM in hexane	32	212	202.51–205.55	1.534	[a]_D_ −118
2	Cyclo(L-Tyr-L-Tyr)	75% DCM in hexane	44	278.4	265.1–267.34	4.654	[a]_D_ +145
3	Cyclo-(L-Phe-Gly)	15% ethyl acetate in DCM	26	238, 276	262.23–265.58	6.121	[a]_D_ −167
4	Cyclo(4-hydroxy-L-Pro-L-Trp)	35% ethyl acetate in DCM	33	268, 278.4	161.56–165.67	7.823	[a]_D_ −065

### Identification of bioactive compound

**Figure 4:**
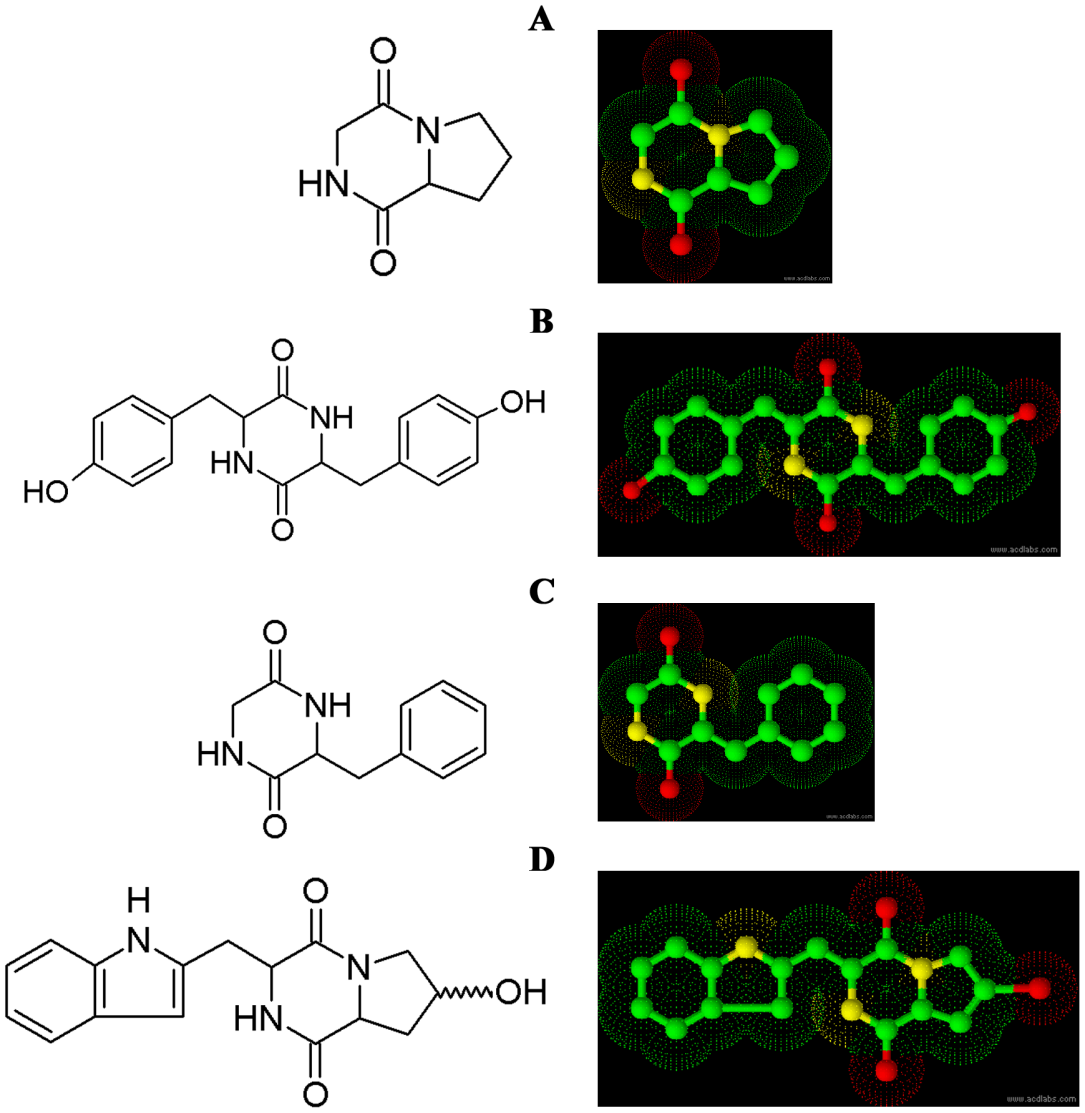
The structure of diketopiperazines. (**A**) Cyclo-(L-Pro-Gly), (**B**) Cyclo(D-Tyr-D-Tyr), (**C**) Cyclo-(L-Phe-Gly) and (**D**) Cyclo(4-hydroxy-L-Pro-L-Trp).

**Figure 5:**
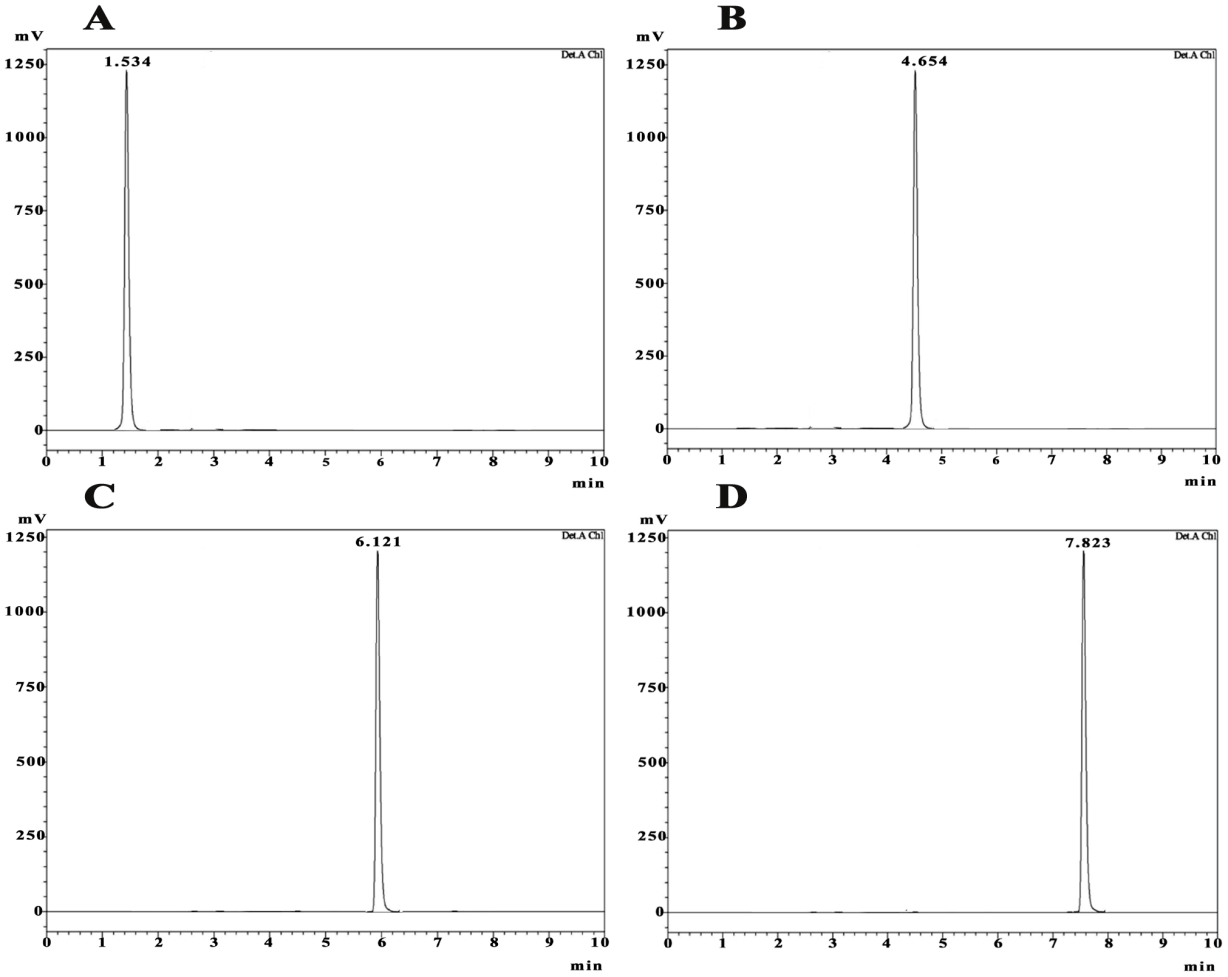
HPLC chromatogram of diketopiperazines on a reversed-phase C18 column (LC-20AD). Samples of 15 µl were injected to a column (250 mm×4.6 mm×5 mm), eluted with 100% methanol. Retention time is 2.778 min. The calculated purity is 96% based on the peak area. (**A**) Cyclo-(L-Pro-Gly), (**B**) Cyclo(D-Tyr-D-Tyr), (**C**) Cyclo-(L-Phe-Gly) and (**D**) Cyclo(4-hydroxy-L-Pro-L-Trp).

The pure compounds were subjected to various spectroscopic analyses, i.e. UV, NMR and HRMS. The structure of compounds corresponded to a four different diketopiperazines (DKPs). The DKPs identified were cyclo(L-Pro-Gly), cyclo(D-Tyr-D-Tyr), cyclo(L-Phe-Gly) and cyclo(4-hydroxy-L-Pro-L-Trp) (Fig. 4). The melting point, HPLC retention time and optical rotation of the compounds are shown in Table 2. In HPLC analysis the purity of the compounds reached greater than 96% according to the peak area (Fig. 5).

#### DKP 1: Cyclo-(L-Pro-Gly); hexahydropyrrolo[1,2-a]pyrazine-1,4-dione was obtained as white crystals

The spectral data of DKP 1 (see File S1). The spectral data were in agreement with those reported earlier in literature [29].

#### DKP 2: Cyclo(L-Tyr-L-Tyr); 3,6-bis(4-hydroxybenzyl)piperazine-2,5-dione was obtained as colorless solid

The spectral data of DKP 2 (see File S1). These data were similar to those previously reported [30].

#### DKP 3: Cyclo-(L-Phe-Gly); 3-benzylpiperazine-2,5-dione was obtained as white crystal

The spectral data of DKP 3 (see File S1). These data were similar to those previously reported [31].

#### DKP 4: Cyclo(4-hydroxy-L-Pro-L-Trp); 7-hydroxy-3-(1H-indol-2-ylmethyl)hexahydropyrrolo[1,2- a]pyrazine-1,4-dione was obtained as colorless needles

The spectral data of DKP 4 (see File S1). The spectra were identical to those recorded in literature [32].

### Absolute Configuration Determination of compounds by the HPLC analysis of Marfey’s derivatives

The modified Marfey’s method which successfully applied for determining the absolute stereochemistry of the four compounds. Regarding the absolute stereochemistry, all the compounds contain L amino acids except glycine with is achiral (Figure S1–S4 in File S1).

### Antifungal activity

MIC values of compounds against twelve fungi are provided in Table 3. Compounds recorded antifungal activity against all the tested fungi except DKP 1. DKP 4 recorded significant activity against all test fungi especially against *Aspergillus* species (Table 3). The result of disc diffusion assay by MIC concentration of the compounds is also presented in the Table 3.

**Table 3:** Antifungal activity of diketopiperazines.

Test fungi	DKP 1		DKP 2		DKP 3		DKP 4		Amphotericin B	
	MIC(µg/ml)	Zone of inhibition(Dia. in mm)	MIC(µg/ml)	Zone of inhibition(Dia. in mm)	MIC(µg/ml)	Zone of inhibition(Dia. in mm)	MIC(µg/ml)	Zone of inhibition(Dia. in mm)	MIC(µg/ml)	Zone of inhibition(Dia. in mm)
*A. flavus*	–	–	16	22±0	64	25±0	2	32±0.57	8	24±1.52
*A. niger*	–	–	32	17±0.57	64	27±1	4	29±0	4	31
*A. tubingensis*	–	–	16	19±1.15	125	26±1.15	8	27±0.57	16	16±0
*A. fumigatus*	–	–	8	21±1.15	250	30±1.15	16	24±0.57	32	19±1.52
*A. parasiticus*	–	–	64	23±1.15	250	20±0.57	16	30±0	16	24±1.15
*C. albicans*	–	–	64	15±1	500	24±1	8	22±0.57	2	24±1.52
*F. oxysporum*	–	–	16	22±1.73	–	–	16	29±0.57	16	24±1.73
*R. solani*	–	–	64	21±0	–	–	16	24±0	32	21±1.15
*P. expansum*	–	–	250	11±1.73	32	30±1.73	16	19±1.2	16	25±1.2
*C. gastricus*	–	–	125	22±1	125	33±1.73	32	21±1.2	8	30±1.15
*C. tropicalis*	–	–	125	27±1.73	500	25±1.52	16	25±1	2	23±1.15
*T. rubrum*	500	11±1	250	15±1	64	23±0	4	30±1	4	36±1.73

–no activity up to 1000 µg/ml.

### Application of cyclo(4-hydroxy-L-Pro-L-Trp) from *B. cereus* in peanut food model as biopreservative agent

**Figure 6:**
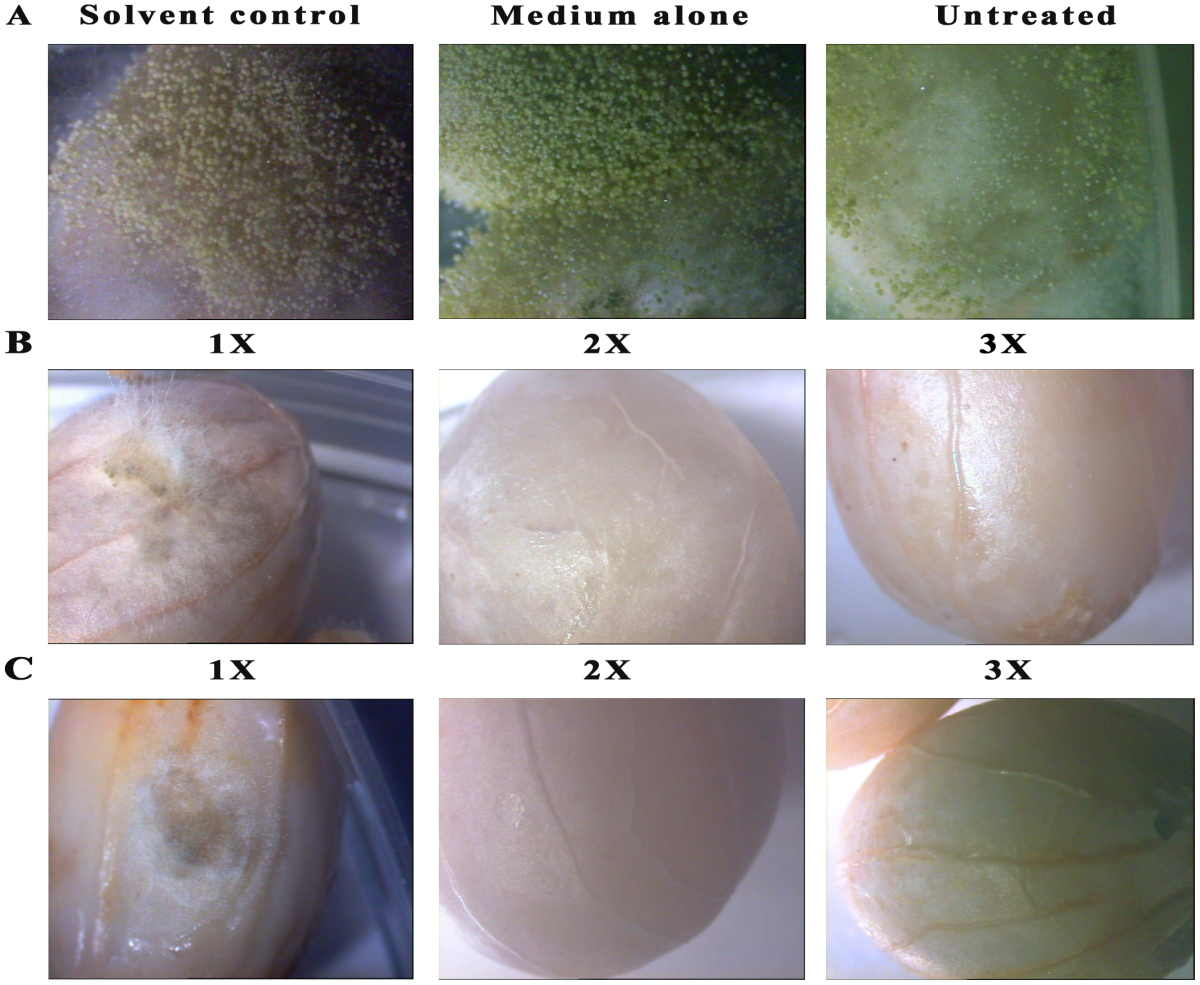
Microscopic images (Carl Zeiss stereomicroscope, Stemi 200C) of the growth of *A. flavus* in control and peanut kernels treated with crude ethyl acetate extract and cyclo(4-hydroxy-L-Pro-L-Trp) after 2 days. (**A**) Control plates: solvent control-treated with methanol, medium alone- treated with autoclaved modified medium and untreated- peanut kernels without methanol and modified medium (**B**) crude extract (**C**) cyclo(4-hydroxy-L-Pro-L-Trp). 1 ×, 2 ×, and 3 × are the 1-fold, 2-fold, and 3-fold MIC concentration of cyclo(4-hydroxy-L-Pro-L-Trp) or crude ethyl acetate extract.

**Figure 7:**
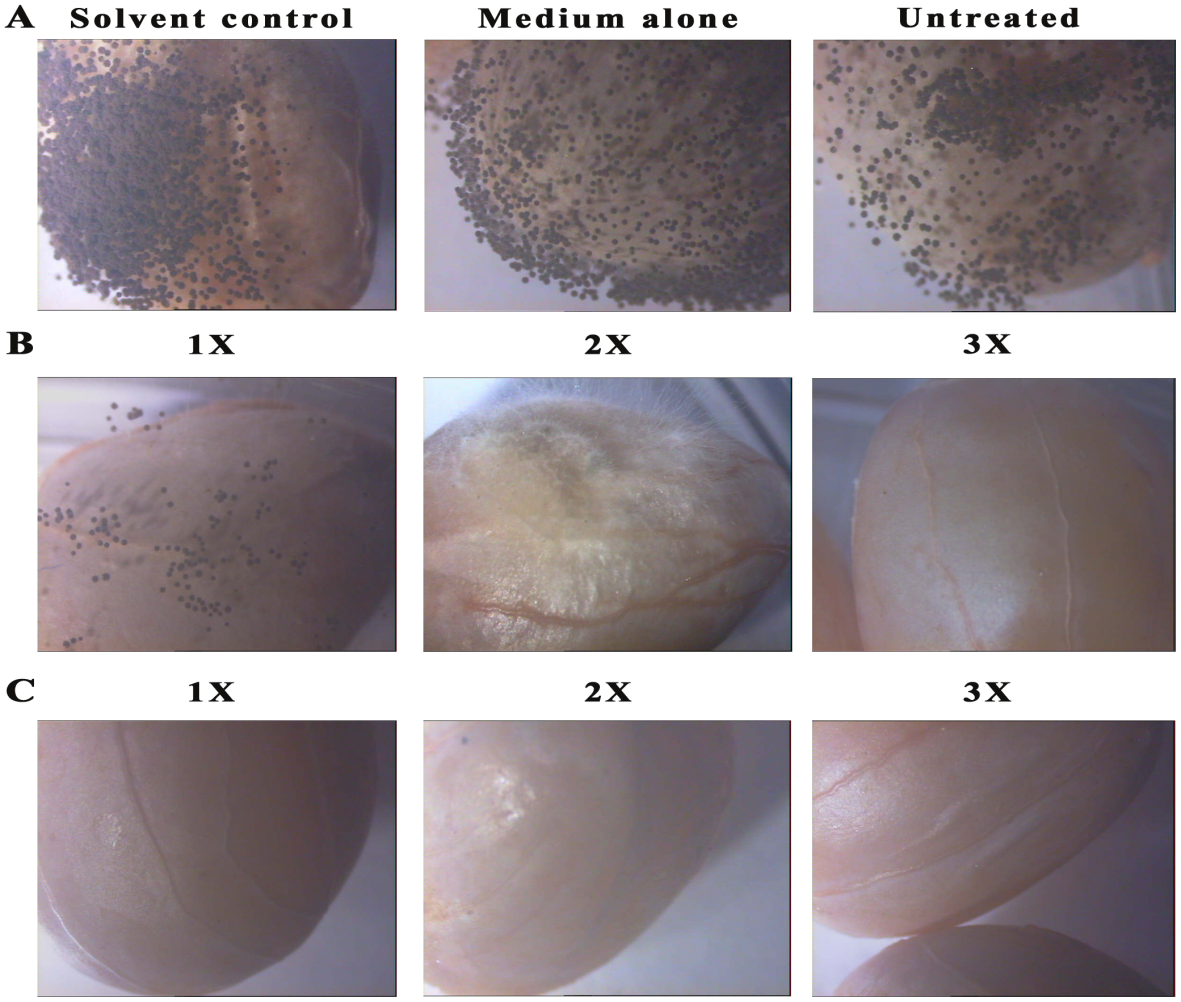
Microscopic images (Carl Zeiss stereomicroscope, Stemi 200C) of the growth of *A. niger* in control and peanut kernels treated with crude ethyl acetate extract and cyclo(4-hydroxy-L-Pro-L-Trp) after 2 days. (**A**) Control plates: solvent control-treated with methanol, medium alone- treated with autoclaved modified medium and untreated- peanut kernels without methanol and modified medium (**B**) crude extract (**C**) Cyclo(4-hydroxy-L-Pro-L-Trp). 1 ×, 2 ×, and 3 × are the 1-fold, 2-fold, and 3-fold MIC concentration of cyclo(4-hydroxy-L-Pro-L-Trp) or crude ethyl acetate extract.

The ability to prevent the growth of *A. flavus* and *A. niger* on stored pulses would be of major significance with regards to health of humans, animals and agricultural economy. For this reason, peanut kernels was used as a food model to investigate the potential application of cyclo(4-hydroxy-L-Pro-L-Trp) to eliminate fungal spoilage in food and feed (Figs. 6 and 7). White mycelia and dark green spores were observed in control peanut kernels for 1 and 2 days after inoculating the *A. flavus* (Fig. 6A). Whereas black spores were observed in control peanut kernels inoculated with *A. niger* after 1 and 2 days (Fig. 7A). As shown in Fig. 6B and 7B (observation after 2 days), the growth of *Aspergillus* species was observed in peanut kernels treated with 1×MIC concentration of crude extract. However, partial growth inhibition was observed in peanuts treated with 2×MIC concentration of crude extract and no growth was observed in peanut kernels treated with 3×MIC concentration of crude extract (Fig. 6B and 7B). As shown in Fig. 6C (observation after 2 days), peanut kernels treated with 1×MIC concentration of cyclo(4-hydroxy-L-Pro-L-Trp) recorded slight *A. flavus* growth, but no fungal growth and spores were observed on peanut kernels treated with 2× and 3× MIC concentration of cyclo(4-hydroxy-L-Pro-L-Trp) (Fig. 6C). However no *A. niger* growth was observed for peanuts treated with 1×MIC concentration of cyclo(4-hydroxy-L-Pro-L-Trp) (Fig. 7C). *Aspergillus* species did not germinate in peanut kernels treated with the 2 and 3-fold MIC concentration of cyclo(4-hydroxy-L-Pro-L-Trp), even after 2 weeks (data not shown).

### Cytotoxicity test

**Figure 8:**
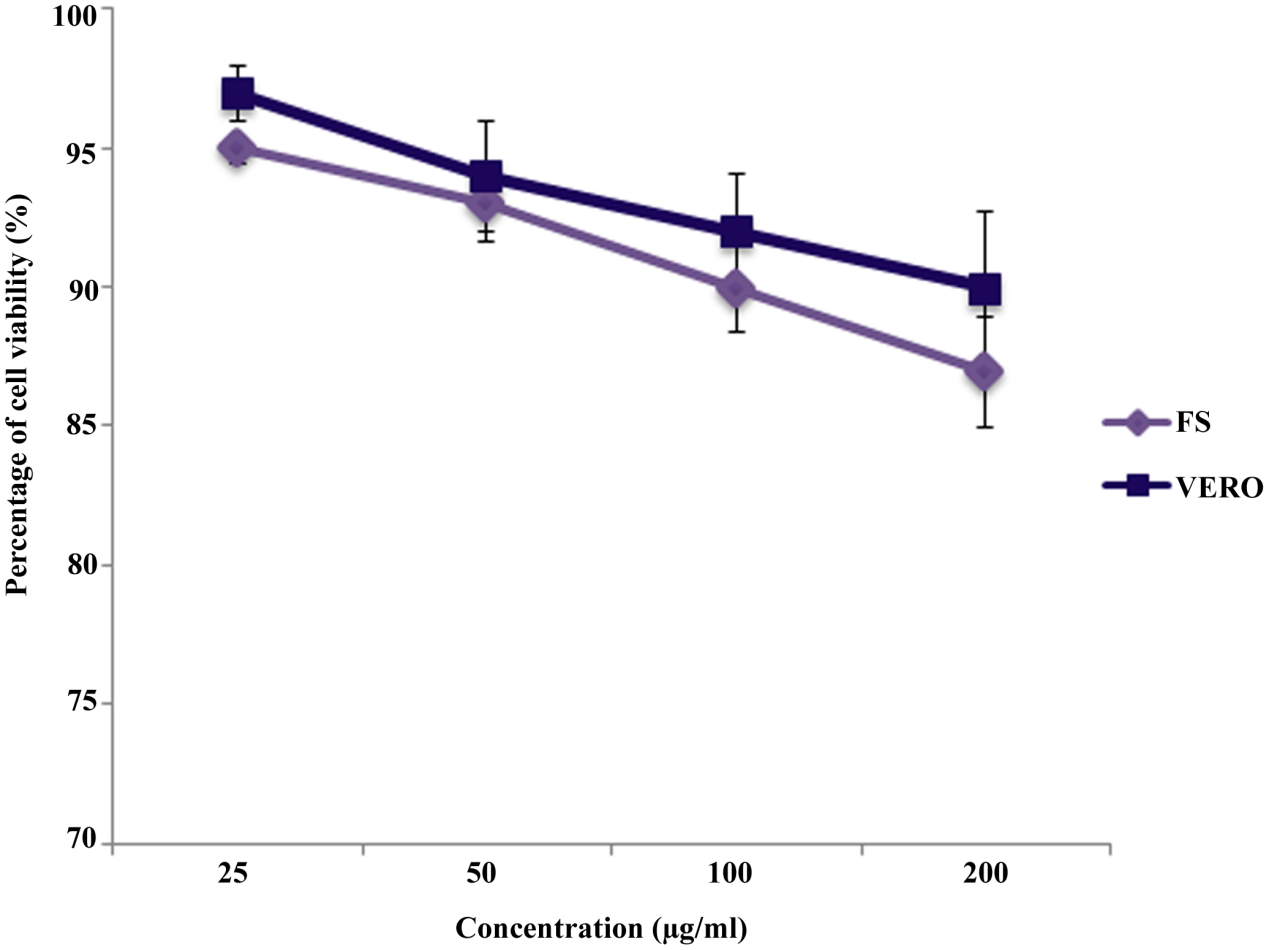
Cytotoxicity of cyclo(4-hydroxy-L-Pro-L-Trp) against normal cell lines.

The cyclo(4-hydroxy-L-Pro-L-Trp) is nontoxic to healthy human and animal cell lines up to 200 µg/ml (Fig. 8). This clearly indicated that cyclo(4-hydroxy-L-Pro-L-Trp) is safe for biopreservative purposes.

## Discussion

A major problem in the storage of foods and feed stocks is spoilage and poisoning caused by fungi, *Aspergillus*, *Fusarium*, *Penicillium* species and causes great economic losses worldwide [33]. Furthermore, these fungi produce allergenic spores and mycotoxins that cause serious potential health hazards [34]. Adequate control measures to prevent fungal growth in grains, foodstuffs, foods and feed production and storage are primary importance to avoid contamination and minimizing public health hazards. During the last few years, there has been a growing interest in biopreservation, i.e., the application of microorganisms and/or their metabolites to prevent spoilage and to extend the preservation time of foods [35].

In recent years, consumers are more concerned about the processed foods they use. Demands for natural, high quality and preservative-free products that are safe and stable introduce a great challenge for the food industry [36]. Currently, there is a strong debate about safety aspects of chemical preservatives since they are considered responsible for many carcinogenic and teratogenic attributes as well as residual toxicity [37]. Some microorganisms have traditionally been used as biopreservatives in food and feed. In recent years, some antagonists have been applied in biocontrol of postharvest diseases of agricultural products. For example, a new strain of *Bacillus pumilus* isolated from Korean soybean sauce showed strong antifungal activity against the aflatoxin-producing fungi *A. flavus* and *A. parasiticus* [38]. *Serratia plymuthica* 5–6, isolated from the rhizosphere of pea, is reported to reduce dry rot of potato caused by *Fusarium sambucinum* [39]. In the present study *B. cereus* associated with EPN recorded significant biocontrol property in reducing the *Aspergillus* species growth in peanut.

The results of the present study demonstrated for the first time that the *B. cereus* has great potential in controlling postharvest disease caused by *Aspergillus* species on peanut kernels. In the *in vitro* tests, 1×10^8^ CFU/ml *B. cereus* cell suspension and bacterial cell free culture filtrate suppressed *Aspergillus* growth. Significant inhibition was recorded for bacterial cell free culture filtrate. Moreover, the percentage of inhibition of bacterial cell free culture filtrate and cell suspension (1×10^8^ CFU/ml) could influence the growth of the pathogen on PDA plates. Thus, we concluded the cells of *B. cereus* had some inhibitory effect on *Aspergillus* species, but the metabolites from the cell free culture filtrate of *B. cereus* recorded significant inhibition to *Aspergillus* species on PDA plates. This finding suggested that *B. cereus* could inhibit the *Aspergillus* species due to some toxic compounds accumulated in the culture medium or antibiotic production. This result was in agreement with that reported from other antagonists such as *B. subtilis* [40] and *B. pumilus* [41].

The results from *in vivo* test also recorded that 1×10^8^ CFU/ml *B. cereus* cell suspension was very much effective in controlling *Aspergillus* species and better control was obtained with longer incubation time. The concentrations of antagonist had significant effects on biocontrol effectiveness: the higher the concentration of *B. cereus*, the better biocontrol activity. The significant biocontrol was obtained at 1×10^1^°CFU/ml, because the disease incidence was lower when compared with other concentrations. These results indicate that, apart from the production of antimicrobial substances by *B. cereus*, bacterial competition for space and nutrition is perhaps another mode of action [20], [42].

Based on the spectral data, we identified the antifungal compounds as cyclo-(L-Pro-Gly), cyclo(D-Tyr-D-Tyr), cyclo-(L-Phe-Gly) and cyclo(4-hydroxy-L-Pro-L-Trp). To our best knowledge, this is the first report on the antifungal activity of EPN bacteria associated cyclo-(L-Pro-Gly), cyclo(D-Tyr-D-Tyr), cyclo-(L-Phe-Gly) and cyclo(4-hydroxy-L-Pro-L-Trp), a 2,5-diketopiperazines (DKPs). These compounds recorded significant antifungal activity against five *Aspergillus* species and other medically important fungi. Recently we reported the antifungal activity of cyclo(L-Pro-L-Leu), cyclo(D-Pro-L-Leu), and cyclo(L-Pro-D-Leu) from TSB medium against pathogenic fungi and bacteria [43].

DKPs are the smallest possible cyclic peptides composed of two α-amino acids. They are abundant natural compounds produced by various bacteria like *Streptomyces* species [44], *Pseudomonas aeruginosa* [45], or *Lactobacillus plantarum* [46], fungi, e.g., *Aspergillus flavus* [47] or *Alternaria alternata* [48], and marine sponges like *Dysidea herbacea* [49]. Recently, the interest in this substance class has increased due to their immense bioactivities including antibacterial activity [50], antifungal function [48], cytotoxicity [46], phytotoxicity [48], and inhibition of plasminogen activator inhibitor-1 [51]. DKPs were shown to act as quorum sensing molecules; e.g., cyclo(L-Pro-L-Tyr), was identified in culture supernatant of *Pseudomonas aeruginosa* and was identified as an activator of an *N*-acylhomoserine lactone biosensor [45]. Due to their chiral, rigid, and functionalized structures, they bind to a large variety of receptors with high affinity, giving a broad range of biological activities [52]. The wide spectrum of their biological properties points to various therapeutic possibilities.

From the present study it is very clear that cyclo(4-hydroxy-L-Pro-L-Trp) can inhibit the growth of *A. flavus* and *A. niger* in *in vitro* and *in vivo* conditions and thus it can prevent food spoilage caused by *Aspergillus* species. Cyclo(Phe-Pro) and cyclo(Phe-OH-Pro) produced by the *Lactobacillus coryniformis s*ubsp. *coryniformis* Si3 strain having antifungal property against *Aspergillus* species was also reported earlier [33]. Cyclo(L-Pro-L-Leu) produced by *Achromobacter xylosoxidans* can inhibits aflatoxin production by *Aspergillus parasiticus* has also reported previously [53]. Yan and Chang [27] reported about the biopreservative property of cell-free supernatants of *Lactobacillus plantarum* in soybean treated with *A. flavus* and identified that biopreservative property is due to cyclo(Leu-Leu) present in the cell-free supernatant. However no data was found on the biopreservative property of pure cyclo(Leu-Leu) in the literature. But in our study we did proved the biocontrol and biopreservative property of *B. cereus*, cell free modified media and pure cyclo(4-hydroxy-L-Pro-L-Trp) in peanut kernels for controlling the decay caused by *Aspergillus* species and proved the efficacy of cyclo(4-hydroxy-L-Pro-L-Trp) as a significant biopreservative compound. We have recently reported the antifungal activity of cyclo(L-Pro-D-Leu), from trypticase soy broth (TSB) medium which can be used for controlling the growth of *A. flavus* and *A. niger* in peanut and soybean [54]. In the present study, modified medium recorded significant antifungal activity than the TSB medium (Figure S5 in File S1). Moreover cyclo(4-hydroxy-L-Pro-L-Trp) purified from modified medium also recorded significant activity than cyclo(L-Pro-D-Leu) from TSB medium. This clearly indicates that modified medium is superior to TSB medium in producing antifungal compounds. In addition, biopreservative property of cyclo(4-hydroxy-L-Pro-L-Trp) in peanut kernels against *Aspergillus* species is reported for the first time.

In conclusion, our results showed that the *B. cereus* associated with EPN has potential biocontrol activity against *Aspergillus* species. This potential may extend to direct use in the market to prolong shelf life, provided the antagonist and its metabolites are safe for human consumption. The use of cyclo(4-hydroxy-L-Pro-L-Trp) to prevent fungal of peanuts has interesting potential applications. Further evaluation of cyclo(4-hydroxy-L-Pro-L-Trp) may lead to useful biopreservation systems which can prevent fungal spoilage and mycotoxin formation in food and feed systems. Other compounds produced *B. cereus* recorded promising antifungal property against medically important fungi, which may receive great benefit to pharma industry in near future.

## Supporting Information

File S1 **Detailed spectral data of compounds.**
(DOC)
