# Supplementary material for: Biocontrol of Aspergillus Species on Peanut Kernels by Antifungal Diketopiperazine Producing Bacillus cereus Associated with Entomopathogenic Nematode
Source: PLoS One. 2014 Aug 26;9(8):e106041. doi: 10.1371/journal.pone.0106041 (PMC4144970; doi:10.1371/journal.pone.0106041)
Supplement: File S1 — Detailed spectral data of compounds. (DOC) [file pone.0106041.s001.doc]

**Supplementary data**

**Biocontrol of *Aspergillus* spp. on peanut kernels by antifungal diketopiperazine producing *Bacillus cereus* associated with entomopathogenic nematode**

Sasidharan Nishanth Kumar, Sreerag Ravikumar Sreekala, Dileep Chandrasekaran, Bala Nambisan^*^, Ruby John Anto

S. Nishanth Kumar, Bala Nambisan, Sreerag RS

Division of Crop Protection/ Division of Crop Utilization, Central Tuber Crops Research Institute, Sreekariyam, Thiruvananthapuram 695017, India.

Dileep C

Department of Botany, SD College,

Kalarcode, Thookkukulam, Alappuzha, India

Ruby John Anto

Integrated Cancer Research Program, Division of Cancer Research, Rajiv Gandhi Centre for Biotechnology, Thiruvanathapuram, India

^*^Corresponding author.

Principal scientist

Division of Crop Protection, Central Tuber Crops Research Institute, Sreekariyam, Thiruvanathapuram 695017, India. Tel.: 0471-2598551 Extn. 214; Fax: (0091) 471-2590063

*E-mail address*: balactcri@gmail

**SUPPLIMENTARY FILE S1**

**Spectral data of compounds**

**DKP 1:** **Cyclo-(L-Pro-Gly);** **hexahydropyrrolo[1,2-a]pyrazine-1,4-dione**: was obtained as white crystals; ^1^HNMR (500 MHz, DMSO-d6) δ 4.14 (1H, brt, *J*=7.0 Hz), 4.01 (1H, d, *J*=16.5 Hz), 3.50 (1H, dd, *J*=4.0, 16.5 Hz), 3.40 (2H, m), 2.16 (1H, m), 1.87 (1H, m), 1.81 (2H, m); ^13^CNMR (125 MHz, DMSO-d6): δ 169.8, 164.5, 58.5, 46.1, 45.0. 28.3, 22.7. HRMS [M+H]^+^ C_7_H_10_N_2_O_2_ calcd. for *m/z* 154.07423, found 154.07431.

**DKP 2: Cyclo(L-Tyr-L-Tyr); 3,6-bis(4-hydroxybenzyl)piperazine-2,5-dione.** was obtained as colorless solid. ^1^H NMR (500 MHz, DMSO-d6): δ 8.15 (NH, br.s), 7.36 (1H, dd, *J* = 6.5, 2.0 Hz), 7.13 (1H, dd, J = 6.5, 2.0 Hz), 3.30 (1H, dd, *J* = 13.5, 7.5 Hz), 2.75 (1H, dd, *J* = 13.5, 7.5 Hz), 2.44 (1H, dd, *J* = 7.5, 7.5 Hz). ^13^C NMR (125 MHz, DMSO-d6): δ 167.9, 158.3, 131.4, 127.9, 115.9, 44.2, 40.5. HRMS [M+H]^+^ C_18_H_18_N_2_O_4_ calcd. for *m/z* 326.12665, found 326.12670.

**DKP 3: Cyclo-(L-Phe-Gly); 3-benzylpiperazine-2,5-dione.** was obtained as white crystal. ^I^H NMR (500 MHz, DMSO-d6): δ 8.09 (1H, brs), 7.82 (1H, brs), 7.18–7.28 (5H, m), 4.05 (1H, m), 3.35 (1H, dd, *J* = 17.5, 2.5 Hz), 3.17 (1H, dd, *J* = 13.0, 5.5 Hz), 3.03 (1H, dd, *J* = 13.0, 5.0 Hz), 2.80 (1H, d, *J* = 17.5 Hz); ^13^C NMR (DMSO-*d*6, 125 MHz): δ 167.0, 165.4, 135.2, 129.4, 127.9, 126.5, 55.7, 43.6, 39.2. HRMS [M+H]^+^ C_11_H_12_N_2_O_2_ calcd. for *m/z* 204.08988, found 204.08995. **DKP 4: Cyclo(4-hydroxy-L-Pro-L-Trp); 7-hydroxy-3-(1H-indol-2-ylmethyl)hexahydropyrrolo[1,2- a]pyrazine-1,4-dione**. was obtained as colorless needles. ^1^H NMR (500 MHz, DMSO-d6) d: δ 10.88 (1H, br s), 7.73 (1H, br s,), 7.56 (1H, d, *J* = 7.2 Hz), 7.27 (1H, d, *J* = 8.1 Hz), 7.18 (1H, m), 7.05 (1H, d, J = 1.2 Hz), 5.09 (1H, br s,), 6.97 (1H, m), 4.20 (1H, m), 4.30 (1H, m), 4.07 (1H, m), 3.27 (1H, m), 3.20 (1H, m), 3.09 (1H, m),3.04 (1H, m), 1.95 (1H, m), 1.63 (1H, m);^13^C NMR (125MHz, DMSO-d6): δ 169.4, 165.8, 135.9, 127.9, 124.5, 121.0, 119.3, 118.7, 111.2, 108.6, 67.6, 56.4, 55.3, 53.7, 36.2, 26.4. HRMS [M+H]^+^ C_16_H_17_N_3_O_3_ calcd. for *m/z* 299.12699, found 299.12687.

**Figure S1.** **HPLC profile of FDAA derivatives of L-Proline, Glycine and Cyclo(L-Pro-Gly) (DKP 1)**

**
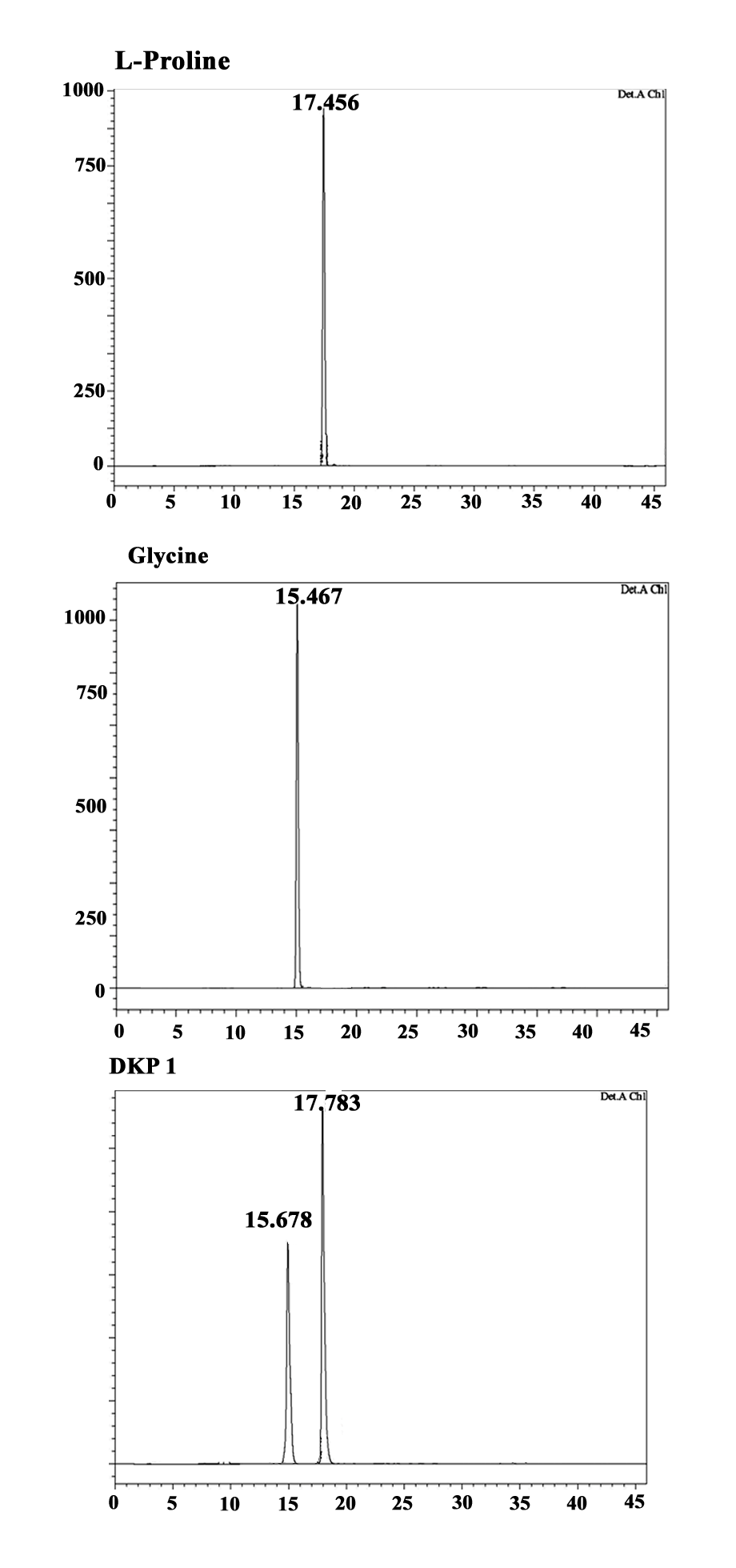
**

**Figure S2.** **HPLC profile of FDAA derivatives of L-Tyrosine, D-Tyrosine and Cyclo(L-Tyr-L-Tyr) (DKP 2)**

**
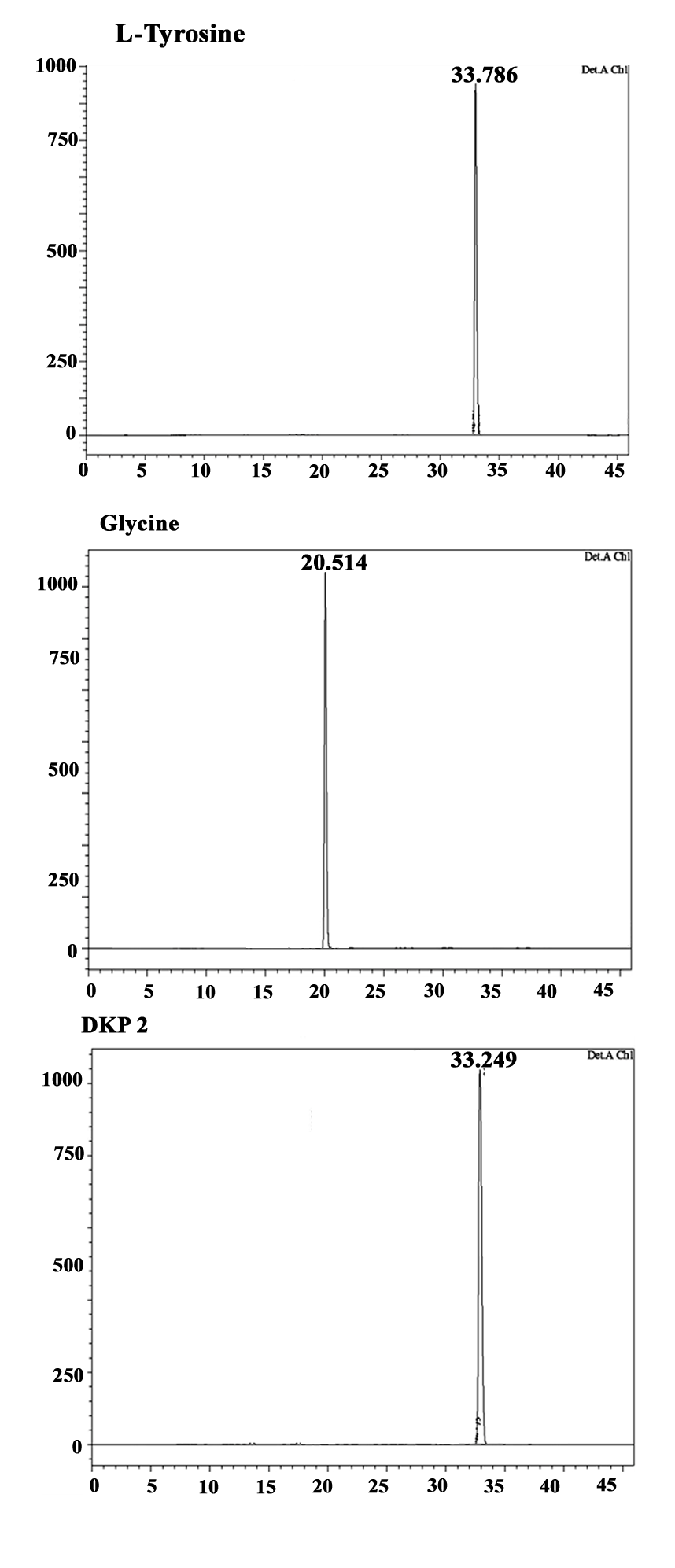
**

**Figure S3.** **HPLC profile of FDAA derivatives of L-Phenylalanine, Glycine and Cyclo(L-Phe-Gly) (DKP 3)**

**
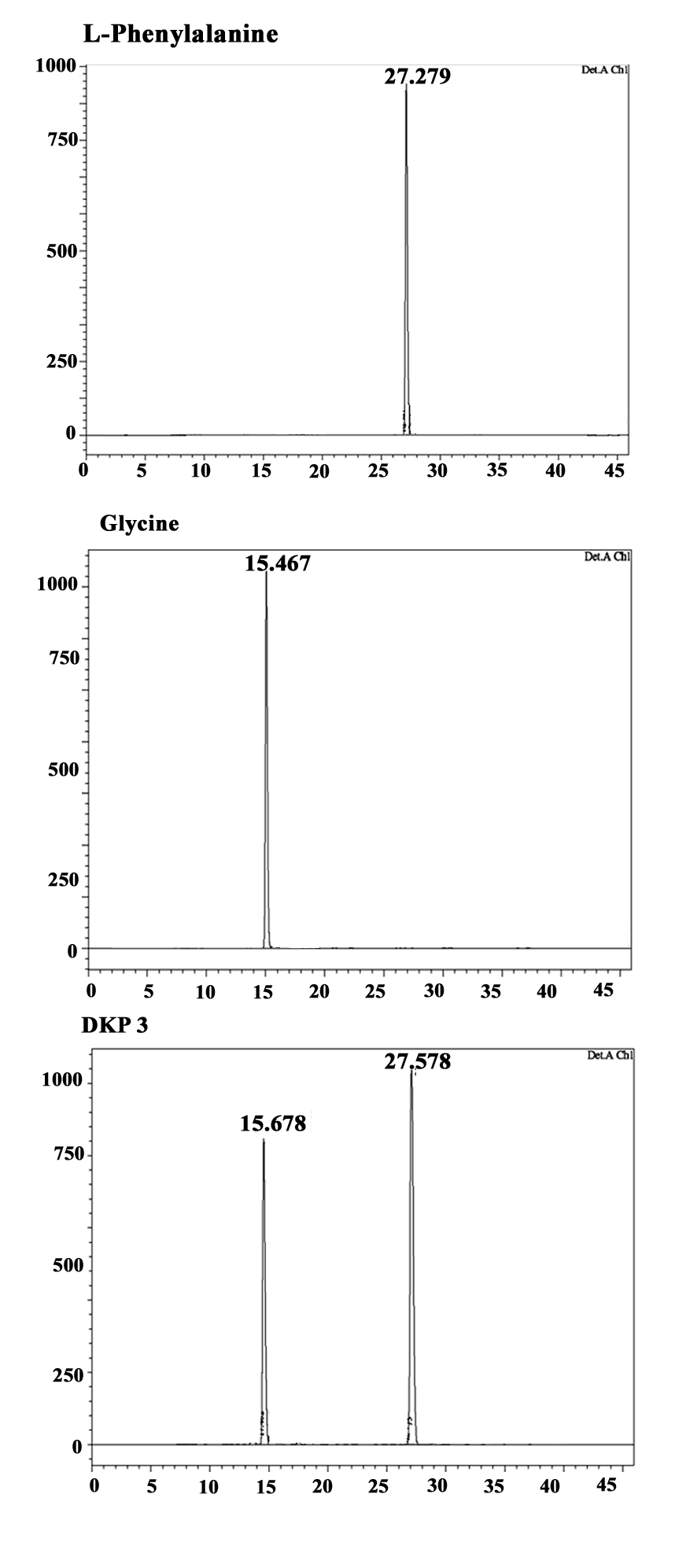
**

**Figure S4.** **HPLC profile of FDAA derivatives of L-Tyrosine, L-4-hydroxy-L-Pro and Cyclo(4-hydroxy-L-Pro-L-Trp) (DKP 4)**

**
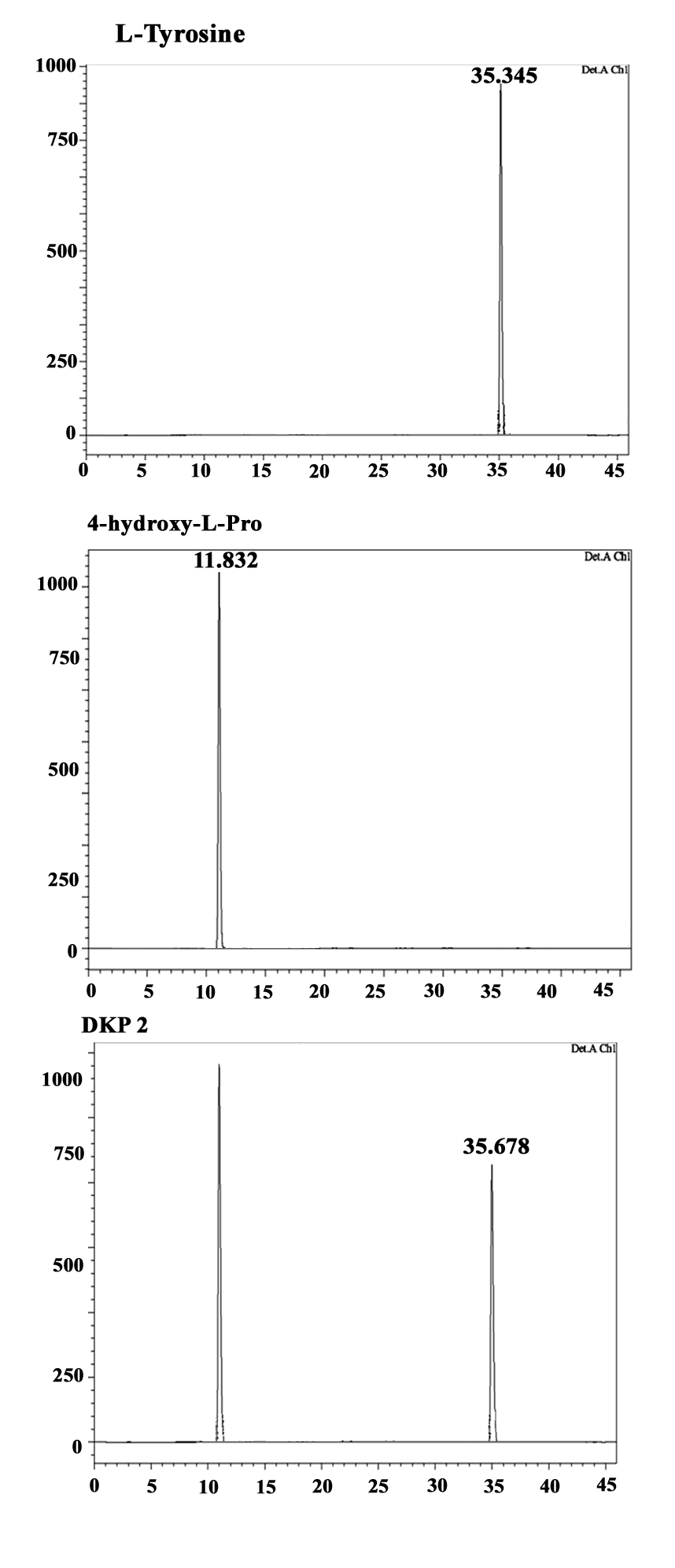
**

**Figure S5: Antifungal activity of modified medium and TSB**

**
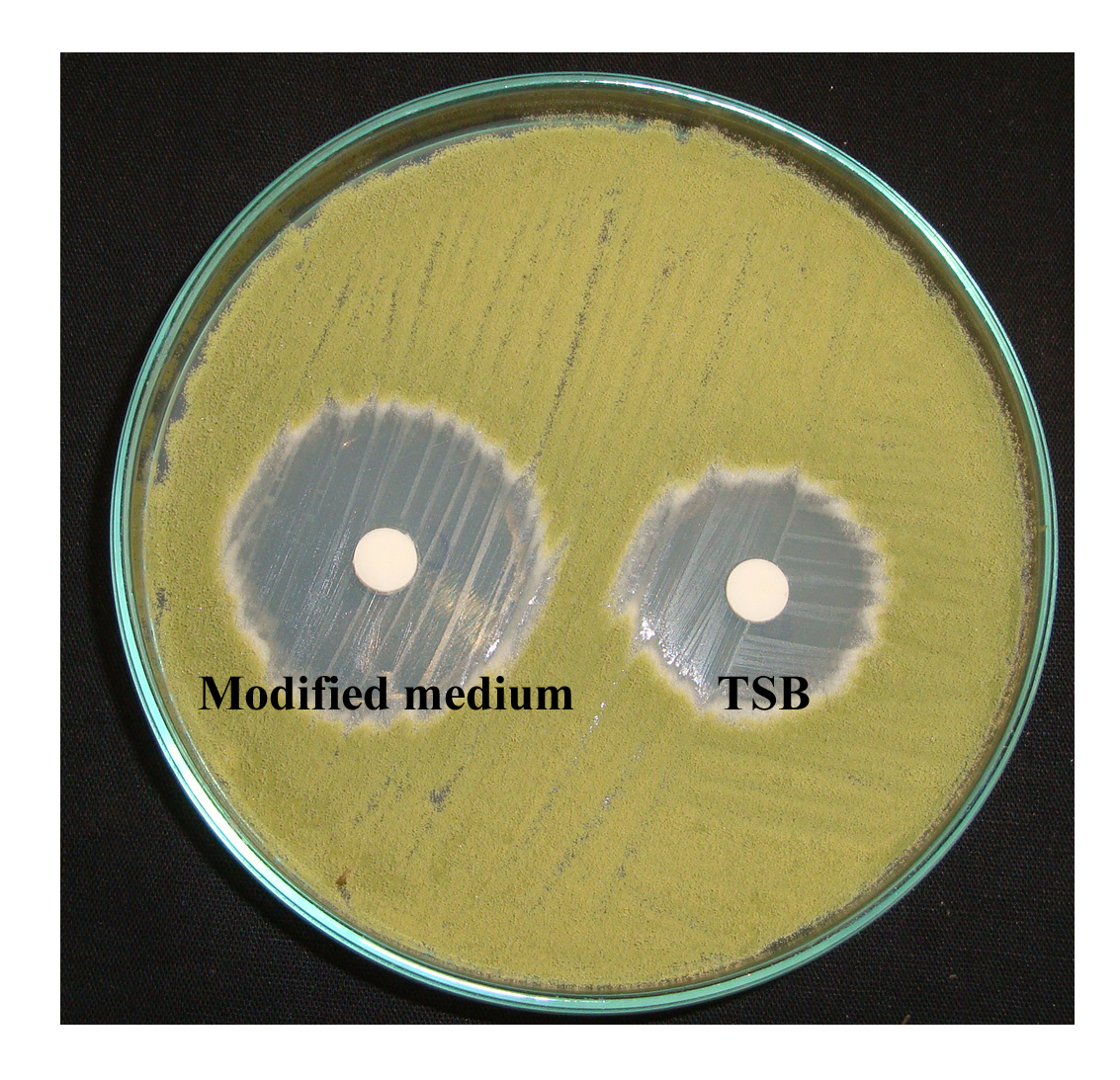
**
